# OvidSP Medline-to-PubMed search filter translation: a methodology for extending search filter range to include PubMed's unique content

**DOI:** 10.1186/1471-2288-13-86

**Published:** 2013-07-02

**Authors:** Raechel A Damarell, Jennifer J Tieman, Ruth M Sladek

**Affiliations:** 1 Palliative and Supportive Services, Flinders University, GPO Box 2100, Adelaide 5001, Australia; 2 Health Professional Education, School of Medicine, Flinders University, GPO Box 2100, Adelaide 5001, Australia

**Keywords:** Information Retrieval, Search Filters, Search Filter Translation, PubMed, Medline, Medical Subject Headings (MeSH)

## Abstract

**Background:**

PubMed translations of OvidSP Medline search filters offer searchers improved ease of access. They may also facilitate access to PubMed’s unique content, including citations for the most recently published biomedical evidence. Retrieving this content requires a search strategy comprising natural language terms (‘textwords’), rather than Medical Subject Headings (MeSH). We describe a reproducible methodology that uses a validated PubMed search filter translation to create a textword-only strategy to extend retrieval to PubMed’s unique heart failure literature.

**Methods:**

We translated an OvidSP Medline heart failure search filter for PubMed and established version equivalence in terms of indexed literature retrieval. The PubMed version was then run within PubMed to identify citations retrieved by the filter’s MeSH terms (*Heart failure, Left ventricular dysfunction,* and *Cardiomyopathy)*. It was then rerun with the same MeSH terms restricted to searching on title and abstract fields (i.e. as ‘textwords’). Citations retrieved by the MeSH search but not the textword search were isolated. Frequency analysis of their titles/abstracts identified natural language alternatives for those MeSH terms that performed less effectively as textwords. These terms were tested in combination to determine the best performing search string for reclaiming this ‘lost set’. This string, restricted to searching on PubMed’s unique content, was then combined with the validated PubMed translation to extend the filter’s performance in this database.

**Results:**

The PubMed heart failure filter retrieved 6829 citations. Of these, 834 (12%) failed to be retrieved when MeSH terms were converted to textwords. Frequency analysis of the 834 citations identified five high frequency natural language alternatives that could improve retrieval of this set (*cardiac failure*, *cardiac resynchronization*, *left ventricular systolic dysfunction, left ventricular diastolic dysfunction*, and *LV dysfunction)*. Together these terms reclaimed 157/834 (18.8%) of lost citations.

**Conclusions:**

MeSH terms facilitate precise searching in PubMed’s indexed subset. They may, however, work less effectively as search terms prior to subject indexing. A validated PubMed search filter can be used to develop a supplementary textword-only search strategy to extend retrieval to PubMed’s unique content. A PubMed heart failure search filter is available on the CareSearch website (http://www.caresearch.com.au) providing access to both indexed and non-indexed heart failure evidence.

## Background

Health professionals need time-efficient access to existing and emerging evidence for effective clinical decision making. However, identifying relevant evidence within large biomedical databases at the point of need can be difficult for even the most experienced searchers due to time pressures and a burgeoning volume of high level evidence, specifically randomised controlled trials and systematic reviews [1]. Search filters that are readily accessible and easy to use may remove some of the barriers clinicians confront in searching large, sophisticated biomedical databases.

### Validated search filters

A search filter is a pre-tested search strategy designed to identify and retrieve a specific subset of literature from a large database. This subset may consist of studies with a particular study design in common (‘methodological filters’) [2-4] or articles on a specific topic (‘topic filters’) [5-8]. Search filters that have been developed using a transparent and robust empirical method are arguably more trustworthy than those based on expert opinion without a testing process [9,10]. These ‘validated’ search filters characteristically employ a test set of relevant citations carefully chosen to minimise the chance of biasing filter effectiveness. This test set (or ‘gold standard’) provides a basis upon which to iteratively test and improve the search filter, as well as a final metric-based performance estimate that informs prospective users of the proportion of all relevant citations they can expect the filter to retrieve (filter ‘recall’ or ‘sensitivity’) [9]. A further metric called precision can also be determined. Precision is the number of relevant citations retrieved as a proportion of all citations retrieved [9].

Search filters comprise database-specific syntax and usually include search terms derived from a database-specific thesaurus. They are therefore designed for exclusive use in the database and platform within which they were developed. Translating a search filter for application in another database or platform requires a thorough understanding of the target product’s subject coverage, search algorithm, syntactical rules, and thesaurus. Even small search filter translation errors or unaccounted for differences in search rules can result in significant variation in retrieval performance between databases (e.g. Embase and Medline), or even different interfaces to the same database [11]. This suggests it may be more reliable to develop a search filter from scratch within a new database using a gold standard specific to that resource, rather than create an expert translation and extrapolate its level of performance to another database. OvidSP Medline to PubMed translation may prove to be an exception as these databases share a concept thesaurus and a common dataset for a significant proportion of content.

### OvidSP Medline and PubMed

Medline is a major biomedical database produced by the U.S. National Library of Medicine (NLM), containing over 20 million citations from approximately 5,600 unique journal titles [12]. Ovid Technologies provides a proprietary interface to Medline (hereon ‘OvidSP Medline’) which offers a range of advanced search features. Medline is also freely available as the primary component of the NLM’s PubMed system which currently provides access to more than 22 million citations [13]. Both OvidSP Medline and PubMed use the NLM’s controlled vocabulary thesaurus for indexing articles for Medline (MeSH). This degree of overlap between the two databases makes it feasible to develop a search filter using the OvidSP interface (often preferred by filter developers because its content is more static during the process of filter development) and then reliably and accurately translate it for application in PubMed.

### Why translate for PubMed?

To support clinicians providing end-of-life care to heart failure patients, researchers with the CareSearch project recently developed and validated a heart failure search filter for use in OvidSP Medline [7]. This search filter demonstrates 98% sensitivity and 75% precision within this database. A translation of this filter for PubMed was planned at the outset for two reasons of prime importance to the knowledge translation process: ease of access to evidence, and the maximal timeliness of that evidence.

### Ease of access

PubMed offers free, open access to Medline, making it possible to convert a search filter’s complex search strategy into a URL hyperlink. This URL hyperlink can then be embedded in any webpage, enabling users to launch a real-time PubMed search by simply clicking on it [14]. In contrast, the ability to use an OvidSP Medline search filter is contingent on the user having access to the database via a paid licence at an institutional level and the user faithfully reproducing the filter and saving it in a personalised account for further use.

### Timeliness of evidence

PubMed offers searchers more timely access to the new and emerging evidence in a field than OvidSP Medline. At any point in time, 2% of PubMed’s citations are unique to PubMed [13]. Furthermore, by far the largest proportion of this unique content comprises citations to recently published research articles, submitted electronically to NLM by journal publishers. The NLM assigns these citations the ‘as supplied by publisher’ status tag while they await assessment to determine if they are on topics that lie within the scope of Medline. If they are, their status tag will eventually change to ‘in process’ upon which they will become accessible through OvidSP’s *MEDLINE In-Process* &*Other Non-Indexed Citations* database in addition to PubMed.

Other citations unique to PubMed include those for articles with full-text in PubMed Central (PMC). This content will be missed if searching outside of PubMed.

### Accurate OvidSP Medline to PubMed translation

To be confident in a PubMed translation of an OvidSP Medline search filter, it is important to demonstrate empirically that both versions have an equivalent level of performance in their respective databases. A straightforward methodology for establishing retrieval equivalence between OvidSP Medline and PubMed versions of the same search filter was adhered to [15]. This method involves the following steps:

1. Recreating the full gold standard set of citations used to develop and validate the OvidSP Medline heart failure filter within both the OvidSP Medline and PubMed databases (n = 876).

2. Running the OvidSP Medline search filter within the OvidSP gold standard set to determine the set of citations this version retrieves (n = 855).

3. Replicating this retrieved set with PubMed using unique identifier numbers.

4. Translating the OvidSP Medline heart failure filter for PubMed by converting OvidSP syntax to PubMed syntax (e.g. the .mp. delimiter converts to [tw] and the / delimiter converts to [mh:noexp])

5. Running the PubMed translation in the full gold standard set saved in PubMed to determine the set of citations the translation retrieves (n = 855).

6. Within PubMed, comparing the set of citations retrieved by the PubMed translation with those previously retrieved by the OvidSP Medline filter to determine if both versions retrieve the same set of citations (i.e. #1 NOT #2 and vice-versa).

### Retrieving PubMed’s unique content

As PubMed’s unique citations do not include MeSH terms from the NLM’s controlled vocabulary thesaurus, they can only be retrieved using author words and phrases occurring in their titles and abstracts (hereon ‘textwords*’*). A PubMed search filter will therefore need to incorporate a textword-only strategy if PubMed’s emerging or unique, non-indexed literature is to be retrieved.

The validated PubMed translation itself can be used as the basis for creating and testing a supplementary search strategy for identifying PubMed’s additional content. This strategy, restricted to the non-indexed subset, can then be combined with the translated component for extended retrieval across the full PubMed database.

Textwords within the validated search filter are already known to be highly effective title/abstract retrieval terms, having been identified as such during the clinician review, frequency analysis, testing, and validation stages of filter development. It may therefore be assumed that they will have an equivalent level of performance when restricted to searching on PubMed’s unique, non-indexed content. The search filter’s high frequency, high performance MeSH terms, however, cannot be simply converted to textwords for inclusion in the textword-only strategy without investigating their relative effectiveness as textwords. MeSH terms may not reflect the natural language used by authors to describe their articles in citation form.

The MeSH term *Neoplasms* is a key example. All citations on the topics of ‘cancer’ or ‘tumours’ within the Medline database are indexed with *Neoplasms.* Searching on this single controlled MeSH term therefore eliminates the need for searchers to construct multi-term searches in an attempt to anticipate the many different ways the same concept may be described by different authors. However, as of October 2012, only 2.5% of PubMed citations indexed with *Neoplasms*, also contain this term in their titles or abstracts. In other words, the term ‘neoplasms’ is rarely used in common parlance to describe cancer. Its inclusion in a textword-only search strategy would result in a failure to retrieve the vast majority of citations on the topic. Similarly, articles employing new terminology to describe emerging concepts may, on occasion, continue to be indexed with imprecise, out-dated MeSH terms for a considerable period of time until more specific, up-to-date MeSH terms are established by the NLM. One example is ‘multimorbidity’ which continues to be indexed with the related but non-equivalent term *Comorbidity *[16]. In some circumstances, the appropriate MeSH term for a topic may be impossible to anticipate, such as the use of *Oceanic Ancestry Group* to describe Australian Aboriginal Peoples. Currently, if this MeSH term were searched as a textword, it would not retrieve a single citation. Searchers must rely on alternative terms from natural language to find current, non-indexed literature on this topic.

### Objectives

This study sought to develop and test an innovative methodology for identifying textword predictors for MeSH terms in advance of MeSH indexing in PubMed. Specifically the study sought to:

1. Analyse the relative efficacy of heart failure search filter MeSH terms when restricted to performing as textwords

2. Identify high frequency textword alternatives for those MeSH terms that demonstrably fail to retrieve an adequate proportion of non-indexed literature when limited to searching on PubMed’s title and abstract fields

3. Use these high frequency textwords to create a textword-only search strategy that identifies a contained, rather than comprehensive, set of relevant non-indexed citations.

This resultant textword search string provides an interim strategy for identifying a subset of relevant citations which are inaccessible to the validated search filter prior to subject indexing.

To the best of our knowledge, this systematic and explicit acknowledgement of PubMed’s non-indexed subset constitutes an innovative and incremental addition to existing search filter translation methodology.

## Method

The study design had five phases:

1. Identifying citations retrieved by heart failure search filter MeSH terms.

2. Identifying citations retrieved by heart failure search filter MeSH terms when converted to textwords.

3. Isolating the subset of citations retrieved in phase 1 (MeSH term searching as MeSH terms) but not in phase 2 (MeSH terms searching as textwords). This is referred to as the ‘Lost Set’.

4. Applying frequency analysis to the Lost Set to identify high frequency alternative textwords for MeSH terms that failed to retrieve well when converted to textwords.

5. Testing all alternative textwords identified in phase 4, singularly and in combination, to find the best textword-only search strategy for reclaiming the Lost Set of citations.

### Phase 1: Citations retrieved by heart failure search filter MeSH terms

The four-term OvidSP Medline heart failure search filter and its PubMed translation are shown in Table 1. The [tw] search tag attached to three of the four search terms in the PubMed translation forces a search on the title, abstract and MeSH term fields of the PubMed record. The [mh:noexp] search tag attached to the fourth term, *Ventricular dysfunction, Left*, restricts the search to the MeSH term field alone. The PubMed translation is therefore empowered to retrieve any citation containing at least one of its four search terms in the MeSH term field (although *left ventricular ejection fraction* cannot be retrieved based on MeSH as it has no MeSH term equivalent).

**Table 1:** Heart failure search filters

***OvidSP medline version***	***PubMed translation***
Heart failure.mp. OR Ventricular dysfunction, Left/ OR cardiomyopathy.mp. OR left ventricular ejection fraction.mp.	Heart failure[tw] OR Ventricular dysfunction, Left[mh:noexp] OR cardiomyopathy[tw] OR left ventricular ejection fraction[tw]

Note: The / and [mh:noexp] search delimiters indicate an unexploded MeSH term search. The .mp. and [tw] search delimiters indicate a search on title, abstract, and MeSH term search fields. Both *Heart failure* and *cardiomyopathy* exist as subject headings in the MeSH thesaurus, therefore these terms search as MeSH terms. *Left ventricular ejection fraction* has no subject heading equivalent and therefore does not work as MeSH term search.

The PubMed heart failure search filter was run in the PubMed database on 21 April 2011 to identify citations retrieved by the filter’s MeSH terms. All non-indexed citations were eliminated from the set by applying the Medline subset limit. Additional limits of English language, ‘has abstract’, and the publication date ‘2010’ were applied to produce a results set of manageable size. To reflect natural word order, the inverted MeSH term *Ventricular dysfunction, Left* was changed to *left ventricular dysfunction*. This change does not impair or negate the term’s MeSH-based retrieval as PubMed maps the *left ventricular dysfunction* textword search to the inverted MeSH term.

### Phase 2: Citations retrieved by search filter MeSH terms as textwords

The PubMed heart failure search filter was then rerun with all search tags converted to [tiab]. This modification creates a search that effectively mimics one restricted to the non-indexed subset of PubMed where retrieval is based solely on term occurrence in the title and abstract fields.

### Phase 3: Citations lost during MeSH term-to-textword conversion

The set of citations retrieved by the first [tw]/[mh:noexp] search but not the [tiab] one was isolated by the Boolean search #1 NOT #2. These citations constitute those initially retrieved because they contain at least one filter search term in their MeSH field. They were not retrieved by the modified search as the same filter search terms do not appear in their title and/or abstract fields. This ‘Lost Set’ represents the unique PubMed heart failure citations that would be missed by the PubMed translation prior to MeSH indexing.

The search strategy described by phases 1–3 is shown in Table 2.

**Table 2:** PubMed search history for study phases 1–3: creating the Lost Set (run 21 April 2011)

**Search**	**Query**	**Items found**
#3	#1 NOT #2	834
#2	(heart failure[tiab] OR left ventricular dysfunction[tiab] OR cardiomyopathy[tiab] OR left ventricular ejection fraction[tiab]) AND English[la] AND 2010[dp] AND “hasabstract” AND Medline[sb]	5995
#1	(heart failure[tw] OR left ventricular dysfunction[mh:noexp] OR cardiomyopathy[tw] OR left ventricular ejection fraction[tw]) AND English[la] AND 2010[dp] AND “hasabstract” AND Medline[sb]	6829

### Phase 4: Identifying high performing natural language alternatives in the Lost Set

The Lost Set was then used to identify natural language terms that might serve as supplementary search filter terms, extending search filter performance across the full PubMed database.

The Lost Set was divided into the following three subsets:

• citations indexed with the *Heart failure* MeSH term but not containing *heart failure* in their titles or abstracts;

• citations indexed with *Ventricular dysfunction, Left* but not containing *left ventricular dysfunction* in their titles or abstracts*;* and

• citations indexed with *Cardiomyopathy* but not containing *cardiomyopathy* in their titles or abstracts.

The fourth term in the heart failure filter, *left ventricular ejection fraction,* was not investigated in this way as, having no equivalent MeSH term, its retrieval is not affected by the [tw] to [tiab] search syntax modification.

Each subset was exported into its own EndNote library. Titles and abstracts of all records within a subset library were then extracted as a text file and imported into Concordance, a text analysis program [17]. This program converts title and abstract terms into a frequency-ranked list of single terms. Single terms clearly not central to the concept under consideration (e.g. *patients*), or with a frequency of 10 or lower, were removed. The phrasal contexts of the remaining single terms were viewed. All high frequency phrase constructions (i.e. n ≥ 10) were retained in the list providing they were specific enough to the concept concerned. For example, ‘left ventricular systolic dysfunction’ was included in the ranked list of high frequency natural language terms for *Ventricular dysfunction, Left* but not ‘left ventricular’ which is an incomplete concept that may occur in other, less relevant contexts.

Each high frequency term/phrase identified was then searched within the subset from which it derived to establish the number of unique citations it could retrieve from that subset. Terms that could not retrieve more than 5% of their own subset were removed from the candidate term list.

### Phase 5: Testing candidate term retrieval in the full Lost Set

Candidate terms were then searched individually in the full Lost Set (i.e. not just their own subset) to establish how well they closed the gap between what was initially retrieved based on MeSH and then lost when MeSH-based searching was disabled. Terms were entered with the [tiab] search tag and combined with the Lost Set using ‘AND’. The natural language term with the highest retrieval (T1) was automatically chosen for inclusion in the supplementary textword-only search strategy. This term was then combined with each of the remaining candidate terms using ‘OR’ to identify the best performing two-term combination in the Lost Set (T2). The T2 construction was then combined with all remaining candidates to determine the best three-term combination (T3), and so on. The purpose of this strategy was to identify and eliminate terms that could not retrieve anything in addition to a preceding term once combined with it using ‘OR’. For example, *LV dysfunction* and *Left ventricular systolic dysfunction* may both retrieve well individually but may retrieve the same set of citations, making the presence of both unnecessary. This process reveals the degree of correlation between terms and any redundancies.

The final textword search string retrieving the maximal number of citations from the Lost Set became the supplementary textword-only search strategy. This strategy, limited to searching PubMed’s unique content by the addition of ‘NOT Medline[sb]’, is combined with the validated PubMed translation to extend the filter’s retrieval of the heart failure literature.

## Results

### Phase 1: Citations retrieved by heart failure search filter MeSH terms

The PubMed heart failure translation retrieved 6829 citations when all terms were appended with either the [tw] or [mh:noexp] search tags and limited to the Medline subset, English language, ‘has abstract’, and the publication date ‘2010.’ When the search tags were subsequently converted to [tiab], the filter retrieved 5995 citations with all the same limits applied. Of the original 6829 citations retrieved, 834 (or 12%) were no longer retrievable once MeSH field searching was disabled. Therefore, MeSH terms in the PubMed translation fail to retrieve 12% of relevant non-indexed heart failure citations when these terms are restricted to title/abstract field retrieval.

The 834 citations of the Lost Set included 346 citations (41.5%) not retrieved when *heart failure*[tw] was converted to *heart failure*[tiab]. Frequency analysis of these 346 citations identified only two natural language search terms capable of retrieving more than 5% of the heart failure subset. These were *cardiac failure* (n = 33; 9.5%) and *cardiac resynchronization* (n = 30; 8.7%).

The conversion of *left ventricular dysfunction*[mh:noexp] to *left ventricular dysfunction*[tiab] accounted for the majority of citations in the Lost Set (n = 444; 53.2%). Frequency analysis of these citations identified only three terms related to the concept which could retrieve more than 5% of the left ventricular dysfunction subset. These were *LV dysfunction* (n = 29; 6.5%), *left ventricular systolic dysfunction* (n = 29; 6.5%) and *left ventricular diastolic dysfunction* (n = 23; 5.2%).

The conversion of *cardiomyopathy*[tw] to *cardiomyopathy*[tiab] resulted in the loss of 76 citations, constituting 9.1% of the Lost Set. Only *Chagas disease* (n = 14; 18.4%) qualified as a high frequency term in this set. However, as cardiomyopathy is just one of many clinical signs and complications of Chagas disease, the two terms cannot be considered equivalent. For this reason, *Chagas disease* was not considered as a candidate textword search term.

Candidate terms for the supplementary textword-only search strategy are reported in Table 3 along with their frequencies expressed as ‘record occurrence,’ or the number of unique subset records retrieved by each term.

**Table 3:** Highest frequency terms identified in individual subsets and their performance in the full lost set

**Terms**	**Record occurrence in individual subsets**	**Record occurrence in full lost set (N = 834)**
	**N (%)**	**N (%)**
**Heart failure subset (n = 346)**		
Cardiac failure	33 (9.5%)	38 (4.6%)
Cardiac resynchronization	30 (8.7%)	41 (4.9%)
**Left ventricular dysfunction subset (n = 444)**		
LV dysfunction	29 (6.5%)	30 (3.6%)
Left ventricular systolic dysfunction	29 (6.5%)	30 (3.6%)
Left ventricular diastolic dysfunction	23 (5.2%)	23 (2.8%)
**Cardiomyopathy subset (n = 76)**		
Chagas disease	14 (18.4%)	NA

With a recall of 4.9% (41/834), the best performing individual term in the full Lost Set was cardiac resynchronization. The best performing final combination of textword terms was: cardiac resynchronization[tiab] OR cardiac failure[tiab] OR left ventricular systolic dysfunction[tiab] OR LV dysfunction[tiab] OR left ventricular diastolic dysfunction[tiab]. This five-term search could retrieve 157/834 (18.8%) of the Lost Set citations.

## Discussion and conclusions

This study shows that a straight conversion of heart failure search filter search terms into textwords for retrieving PubMed’s unique, non-indexed content would fail to retrieve a proportion (12%) of relevant non-indexed literature prior to MeSH indexing. Five additional terms were identified that strengthened the performance of a supplementary textword-only search strategy for capturing this missed content. Limited to the non-indexed subset of PubMed, this strategy works in conjunction with the validated PubMed heart failure search filter without ever compromising the validated translation’s known level of performance.

### The full PubMed heart failure filter

The full PubMed heart failure filter is shown here (supplementary textword-only component for retrieving non-indexed citations in bold):

((heart failure[tw] OR ventricular dysfunction, left[mh:noexp] OR cardiomyopathy[tw] OR left ventricular ejection fraction[tw]) AND Medline[sb]) OR **((heart failure[tiab] OR left ventricular dysfunction[tiab] OR cardiomyopathy[tiab] OR left ventricular ejection fraction[tiab] OR cardiac resynchronization[tiab] OR cardiac failure[tiab] OR left ventricular systolic dysfunction[tiab] OR LV dysfunction[tiab] OR left ventricular diastolic dysfunction[tiab]) NOT Medline[sb]).**

Only the highest frequency terms were shortlisted for testing in the development stage, whereupon redundant terms (those that could not retrieve Lost Set citations in addition to preceding terms) were eliminated. This process ensures that searchers are presented with a contained, rather than comprehensive, set of unique PubMed citations by favouring search precision over search sensitivity (the proportion of all relevant citations retrieved) within the non-indexed subset of PubMed.

This filter has been made available as a hypertext link on the CareSearch website (http://www.caresearch.com.au) to facilitate automated access to the relevant heart failure literature. To enhance its clinical utility, it has also been combined with 39 expert searches on a range of heart failure subjects such as anaemia, renal insufficiency, cognitive impairment, device deactivation, and self-care [14].

### Methodology assessment

When translating between databases, inherent differences in database structure, syntax and search algorithms need to be understood for optimal retrieval. The existence within PubMed of unique content in addition to Medline content provides a case in point. This study described a methodology for assessing the effect of forcing search filter indexing terms to search within a subset that does not include indexing terms.

Frequency analysis proved a useful and objective strategy for identifying natural language terms that regularly co-occur with MeSH-based filter terms. These alternative terms were then assessed for their ability to retrieve Lost Set citations. Only a small number of these terms occurred with relatively high frequency across the entire Lost Set to warrant consideration for inclusion in the supplementary textword-only search strategy. Even then, these terms combined could only capture 18.8% of the lost citations. This finding highlights the diffuse nature of natural language and the value of controlled vocabulary indexing to database searchers. The fact that many citations in the Lost Set were indexed with *heart failure* but did not include this term in either title or abstract indicates that indexers, with their specialist clinical knowledge and access to full text articles, clearly see beyond terms in titles and abstracts when assigning MeSH terms. Indexing can therefore be seen as a value-added process for improving the retrievability of relevant items.

This study has several potential limitations. Firstly, we chose to exclude citations without abstracts from the analysis. This decision was based on the assumption that an imbalance between the number of title words and number of abstract words could skew the word pool for frequency analysis. Furthermore, search terms of high discriminatory power, beyond those already included in the filter may be more likely to occur in the substantive abstract field than the shorter title field. While it was beyond the scope of this study to investigate the significance of this decision, this methodological issue remains unresolved in the area of filter development. Secondly, the cut-off point of 5% for identifying ‘high frequency’ terms was chosen arbitrarily. Whilst it appears reasonable, it may have inadvertently eliminated some highly specific natural language alternatives.

The purpose of this study was to explicitly acknowledge PubMed’s unique content and provide a systematic, reproducible method for accounting for this content in translating a search filter from OvidSP Medline. It was not our aim to develop an additional high sensitivity/recall ‘search filter’ for capturing this content, rather an empirically tested extension of an already validated filter which works in tandem with this filter to incrementally improve retrieval across the entire PubMed system. Although it was outside the scope of this study, a future study might extend the methodology to ‘validating’ this additional component in the traditional search filter development sense, using a ‘gold standard’ set of relevant and non-relevant citations. This approach would make it possible to provide the standard metrics of search performance such as sensitivity, specificity, and precision.

A future study might also investigate the effect of including search statements that incorporate the AND Boolean operator in order to increase retrieval in the Lost Set. We only included phrase constructs in our search strategy which imposes an adjacency condition on search terms, e.g. ‘left ventricular systolic dysfunction’ or ‘LV dysfunction’. The AND operator might serve to broaden the search without too great a cost to search precision, e.g. Left AND (systolic OR diastolic OR LV) AND ventricular AND dysfunction. The use of truncation may further improve retrieval (e.g. ventric* retrieves on *ventricular*, *ventricle*, and *ventricles*).

Although this present study focuses on the technical aspects of filter translation, it may have benefited from greater clinician input, particularly in the area of natural language term selection. Natural language terms were tested based on a numerical measure of their importance (frequency) rather than a clinical judgement of their significance to the topic of heart failure. Furthermore, introducing additional terms into a search can increase the risk of retrieving irrelevant citations. A formal post-hoc assessment of the relevance of the citations retrieved by each textword in the supplementary search strategy may have provided further information on their suitability for inclusion.

Notwithstanding the above, this research has demonstrated that whilst an OvidSP Medline to PubMed search filter translation may provide equivalent retrieval of indexed articles, retrieval in PubMed can be extended to non-indexed articles. An additional textword-only version of the search filter, developed to retrieve PubMed’s unique content, can be combined with the translated version to create a PubMed search that ‘filters’ the entire PubMed system, and not just a subset thereof, to focus on a topic of interest. The final result then offers searchers appealing benefits such as ease of access, timeliness of citations, and more extensive coverage.

## Competing interests

The authors declare they have no competing interests.

## Authors’ contributions

JT conceptualised the study and its methodology and led the data analysis and interpretation stage. RD acquired, analysed, and interpreted the data and drafted the manuscript. RS contributed significant critical revisions of the manuscript. All authors read and approved the final manuscript.

## Pre-publication history

The pre-publication history for this paper can be accessed here:

http://www.biomedcentral.com/1471-2288/13/86/prepub

## References

[B1] Bastian H Glasziou P Chalmers I Seventy-five trials and eleven systematic reviews a day: how will we ever keep up? PLoS Med 2010 7 9 e1000326 20877712 10.1371/journal.pmed.1000326 PMC2943439

[B2] Glanville JM Lefebvre C Miles JN Camosso-Stefinovic J How to identify randomized controlled trials in MEDLINE: ten years on J Med Libr Assoc 2006 94 2 130 136 16636704 PMC1435857

[B3] White VJ Glanville JM Lefebvre C Sheldon TA A statistical approach to designing search filters to find systematic reviews: objectivity enhances accuracy J Inf Sci 2001 27 6 357 370

[B4] Haynes RB Wilczynski N McKibbon KA Walker CJ Sinclair JC Developing optimal search strategies for detecting clinically sound studies in MEDLINE J Am Med Inform Assoc 1994 1 6 447 458 7850570 10.1136/jamia.1994.95153434 PMC116228

[B5] Sladek R Tieman J Fazekas BS Abernethy AP Currow DC Development of a subject search filter to find information relevant to palliative care in the general medical literature J Med Libr Assoc 2006 94 4 394 401 17082830 PMC1629429

[B6] Moerman CJ Deurenberg R Haafkens JA Locating sex-specific evidence on clinical questions in MEDLINE: a search filter for use on OvidSP BMC Med Res Methodol 2009 9 25 19366443 10.1186/1471-2288-9-25 PMC2678151

[B7] Damarell RA Tieman J Sladek RM Davidson PM Development of a heart failure filter for Medline: an objective approach using evidence-based clinical practice guidelines as an alternative to hand searching BMC Med Res Methodol 2011 11 12 21272371 10.1186/1471-2288-11-12 PMC3037346

[B8] Iansavichus AV Haynes RB Shariff SZ Weir M Wilczynski NL McKibbon A Rehman F Garg AX Optimal search filters for renal information in EMBASE Am J Kidney Dis 2010 56 1 14 22 20231047 10.1053/j.ajkd.2009.11.026

[B9] Jenkins M Evaluation of methodological search filters–a review Health Info Libr J 2004 21 3 148 163 15318913 10.1111/j.1471-1842.2004.00511.x

[B10] Hausner E Waffenschmidt S Kaiser T Simon M Routine development of objectively derived search strategies Syst Rev 2012 1 1 19 22587829 10.1186/2046-4053-1-19 PMC3351720

[B11] Bradley SM Examination of the Clinical Queries and Systematic Review “hedges” in EMBASE and MEDLINE J Can Health Libr Assoc 2010 31 2 27 37

[B12] Fact sheet Medlinehttp://www.nlm.nih.gov/pubs/factsheets/medline.html

[B13] Fact sheet MEDLINE, PubMed, and PMC (PubMed Central): How are they different? http://www.nlm.nih.gov/pubs/factsheets/dif_med_pub.html

[B14] Heart failure PubMed searches http://www.caresearch.com.au/caresearch/tabid/1539/Default.aspx

[B15] Sladek RM Tieman J Applying evidence in the real world: a case study in library and information practice Health Info Libr J 2008 25 4 295 301 19076676 10.1111/j.1471-1842.2008.00778.x

[B16] Smith SM Soubhi H Fortin M Hudon C O'Dowd T Managing patients with multimorbidity: systematic review of interventions in primary care and community settings BMJ 2012 345 e5205 22945950 10.1136/bmj.e5205 PMC3432635

[B17] Concordance 3.3 http://www.concordancesoftware.co.uk/

